# A mesh-based model of liver vasculature: implications for improved radiation dosimetry to liver parenchyma for radiopharmaceuticals

**DOI:** 10.1186/s40658-022-00456-0

**Published:** 2022-04-13

**Authors:** Camilo M. Correa-Alfonso, Julia D. Withrow, Sean J. Domal, Shu Xing, Jungwook Shin, Clemens Grassberger, Harald Paganetti, Wesley E. Bolch

**Affiliations:** 1 Medical Physics Program, College of Medicine, University of Florida, Gainesville, FL 32611 USA; 2 J. Crayton Pruitt Family Department of Biomedical Engineering, University of Florida, Gainesville, FL 32611-6550 USA; 3 Massachusetts General Hospital, Harvard Medical School, Boston, MA 02115 USA; 4 Radiation Epidemiology Branch, National Cancer Institute, Rockville, MD 21704 USA

**Keywords:** Liver, Hepatic vasculature, ICRP computational phantom, Radionuclide *S* values

## Abstract

**Purpose:**

To develop a model of the internal vasculature of the adult liver and demonstrate its application to the differentiation of radiopharmaceutical decay sites within liver parenchyma from those within organ blood.

**Method:**

Computer-generated models of hepatic arterial (HA), hepatic venous (HV), and hepatic portal venous (HPV) vascular trees were algorithmically created within individual lobes of the ICRP adult female and male livers (AFL/AML). For each iteration of the algorithm, pressure, blood flow, and vessel radii within each tree were updated as each new vessel was created and connected to a viable bifurcation site. The vascular networks created inside the AFL/AML were then tetrahedralized for coupling to the PHITS radiation transport code. Specific absorbed fractions (SAF) were computed for monoenergetic alpha particles, electrons, positrons, and photons. Dual-region liver models of the AFL/AML were proposed, and particle-specific SAF values were computed assuming radionuclide decays in blood within two locations: (1) sites within explicitly modeled hepatic vessels, and (2) sites within the hepatic blood pool residing outside these vessels to include the capillaries and blood sinuses. *S* values for 22 and 10 radionuclides commonly used in radiopharmaceutical therapy and imaging, respectively, were computed using the dual-region liver models and compared to those obtained in the existing single-region liver model.

**Results:**

Liver models with virtual vasculatures of ~ 6000 non-intersecting straight cylinders representing the HA, HPV, and HV circulations were created for the ICRP reference. For alpha emitters and for beta and auger-electron emitters, *S* values using the single-region models were approximately 11% (AML) to 14% (AFL) and 11% (AML) to 13% (AFL) higher than the *S* values obtained using the dual-region models, respectively.

**Conclusions:**

The methodology employed in this study has shown improvements in organ parenchymal dosimetry through explicit consideration of blood self-dose for alpha particles (all energies) and for electrons at energies below ~ 100 keV.

## Introduction

Organ dose assessment is an integral component to both the development and regulatory approval of radiopharmaceuticals [1] and to patient-specific optimization of administered activity in radiopharmaceutical therapy [2–4]. Computational methods for organ dosimetry include the MIRD schema (with pre-computed radionuclide *S* values), dose-point or voxel kernels, and direct Monte Carlo radiation transport [1]. In each case, a geometric model of the patient is required either in the form of a whole-body computational human phantom (reference, patient-dependent, or patient-sculpted)^1^ or as a segmented CT image series as acquired during hybrid imaging (SPECT/CT or PET/CT) [5]. In both approaches, organ anatomy is typically restricted to a model of the organ surface (stylized or polygon mesh) or to the organ volume (collection of image voxels). For phantom-based organ dosimetry, the tissues defined within the organ volumes are a homogeneous mixture of tissue parenchyma and blood content [6]. For CT-based organ dosimetry, the Hounsfield unit of each image voxel can be used to account for tissue heterogeneity across the organ, but this approach still does not permit an explicit differentiation of organ blood and organ parenchyma even at the voxel level.

X-rays and gamma rays detected from emissions within a source organ of the patient originate from decay sites of radiopharmaceutical either in the organ’s vascular network or throughout its tissue parenchyma. In phantom-based macroscale organ dosimetry, the radiopharmaceutical is assumed to be uniformly spatially distributed and, in a single-region organ model, this distinction of decay site location is not resolvable. The radiation absorbed dose to the source organ is further assigned as its mean value across these homogenized tissue constituents. While perhaps well justified for photons and higher-energy beta particles, this approach will tend to overestimate parenchymal absorbed dose for those alpha particles and lower-energy beta particles emitted from the radiopharmaceutical during organ blood transit. In CT-based macroscale organ dosimetry, nonuniformities both in the radiopharmaceutical source and in the tissue absorbed dose may be considered at the voxel level. The lack of distinction of decay site—in either blood or organ parenchyma—still remains, however, even for CT-based patient models.

In this study, we address the need for improvements in phantom-based macroscale organ dosimetry through the development of a geometrically explicit model of intra-organ blood vasculature. In this study, we address one of the most vascularized organs in the human body—the liver. We then quantify the potential for dosimetric improvements through explicit accounting of blood decay sites with a focus on the organ’s larger vessels where blood self-dose is prominent for shorter-ranged particles. Due to the complexity of human vasculature and the inherent difficulty associated with reconstructing blood vessels using only medical image datasets, mathematical and numerical models based on functional and physical principles are proposed. One of the more popular models is that based upon constrained constructive optimization (CCO) [7]. In 1999, Karch et al*.* generalized the CCO method to develop arterial trees of organ vasculature in three dimensions [8]. In 2018, Crookston et al*.* [9] applied the CCO method in the construction of a hepatic arterial tree for stimulation studies of the infusion and trapping of Y-90 microspheres during hepatic tumor radioembolization. In 2022, Sauer et al. similarly created a vascular network in the human liver for use in CT imaging studies of hepatic contrast perfusion [10]. Our work proposes a complete vascular network including the hepatic arterial, portal, and venous blood circulation in the livers of the ICRP reference adult male and adult female human computational phantoms [11], with applications for refined dose assessment of liver parenchyma for internal emitters. The approach used is readily extended to other organs of the body.

## Materials and methods

In this study, we have developed a method to generate virtual binary trees of the hepatic arterial, hepatic venous, and hepatic portal venous vasculature within the livers of the adult male and female ICRP reference mesh phantoms. The models are generated by an algorithm based on the main features of the CCO method [7]. During algorithm execution, geometric and hemodynamic parameters are updated each time a new vessel is created, thus ensuring that total blood flow is preserved and that both Poiseuille’s law and Murray’s law are satisfied at all bifurcation sites.

### Description of vascular tree generation

The first stage to create a representative model of human vasculature consists of specifying the organ 3D shape to be perfused and the hemodynamic properties and physical laws from which the trees develop. In the present model, homogenous perfusion of the target volume is achieved through dichotomously branching tree structures. The terminal segments of the tree are required to be uniformly distributed inside the perfused volume, and the blood flow at each terminal is assumed to be identical across the whole vascular tree.

In our model, vessels are represented as straight and rigid cylindrical pipes. Each pipe is characterized by geometrical parameters (radius *R* and length *l*) and hemodynamics parameters (blood flow *Q* and pressures at the ends of the pipe *P*_in_ and *P*_out_). Each tree constructed begins from a ‘root’ segment. This initial segment is created by connecting the ‘entry’ point from which the tree will be developed to the closest random point from a list of uniformly distributed terminal points (*N*_term_) generated inside the volume to be vascularized. The radius of the root segment is computed via Poiseuille’s equation as shown in Eq. (1), assuming a perfusion pressure (${P}_{\mathrm{perf}}$) at the entry point, a terminal pressure (${P}_{\mathrm{term}}$) at the closest random point, and a terminal blood flow (${Q}_{\mathrm{term}}$) in that pipe segment, and a constant blood viscosity (*μ*) [9, 12].

1 $$\Delta P={P}_{\mathrm{bif}}-{P}_{\mathrm{term}}={Q}_{\mathrm{term}}\frac{8\mu l}{\pi {R}^{4}}$$

**Fig. 1:**
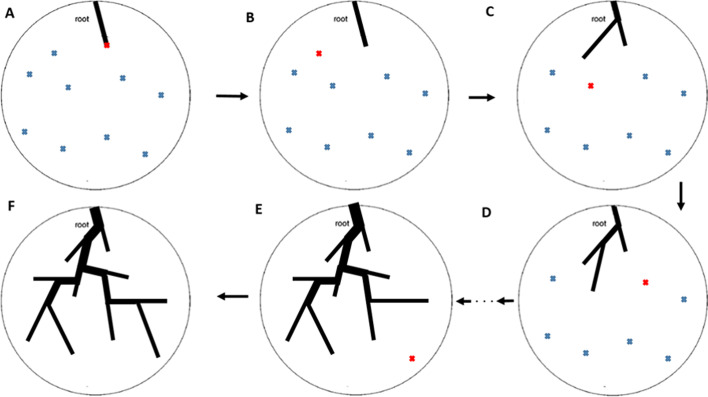
A 2D representation of the in-house vessel generation algorithm developed. **A** A single root segment is created. **B** The closest point (red cross) from the cloud of terminal random points is selected to be added to the existing tree. **C** After the tree is updated, the next closest point to the existing tree is selected. **D, E** The vascular tree is grown by connecting a new pipe to the existing tree and updating all hemodynamics and geometrical parameters. **F** The final tree is constructed, and the algorithm stops when there are no more terminal points to connect to the tree

Once the root segment is created, the tree is generated by subsequently attaching new terminal segments to the existing tree. An overview of the different stages of the algorithm is demonstrated in Fig. 1.

At each iteration, the CCO method chooses the closest random terminal point relative to the center of mass of the existing tree. The midpoint of all segments in the existing tree are evaluated as candidate sites for connection. Later, straight candidate pipes are created from the candidate sites to the chosen terminal point. The same procedure as the one used in the root segment is applied to calculate the radius of each candidate pipe, except the fact that the pressure at the entry of each candidate pipe is equal to the pressure at each candidate site of connections. Finally, the shortest candidate pipe, which is also free of intersections with any other vessel, is chosen and added as a permanent pipe into the tree. Each new permanent pipe added to the existing tree is generated assuming a constant terminal pressure at the pipe end (${P}_{\mathrm{term}}$) and a constant terminal blood flow (${Q}_{\mathrm{term}}$). The radius of the permanent pipe is computed by using Poiseuille’s law [13]. Although blood viscosity $\mu$ depends on several factors [14], above a certain diameter, blood could be considered a Newtonian fluid with constant viscosity. Bezy-Wendling and Bruno [13] state that blood is considered a Newtonian fluid for vessels with radii above 50 $\upmu\mathrm{m}$. In our study, the minimum vessel radius is above 100 $\upmu\mathrm{m}$, and thus a constant blood viscosity of 3.5 mPa-s is considered [12].

**Fig. 2:**
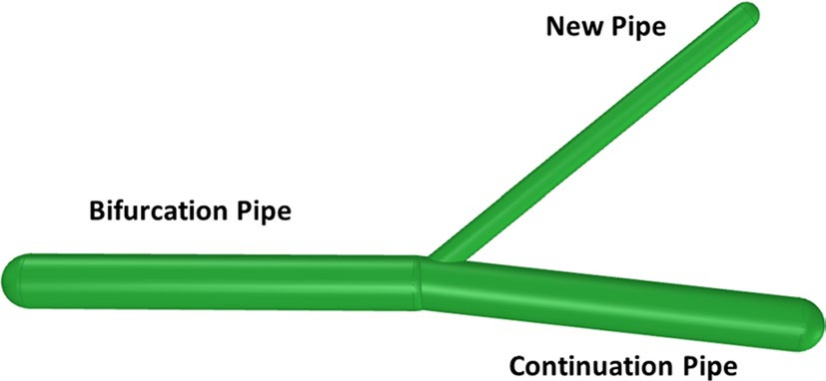
Simplified representation of a bifurcation vessel with two daughters (new and continuation pipes) after the new pipe is permanently added to the virtual tree

When connecting a new pipe to the existing tree, two pipes are created: the new pipe and the continuation pipe, as shown in Fig. 2, with radii ${R}_{\mathrm{new}}$ and ${R}_{\mathrm{con}}$, respectively. The parent of these two pipes is defined as the ‘bifurcation’ pipe. Murray’s law is next applied to compute the radius ${R}_{\mathrm{bif}}$ of the bifurcation pipe. The ‘bifurcation’ exponent $\gamma =3$ was chosen in our study as proposed by Murray [15].

2 $${R}_{\mathrm{bif}}^{\gamma }={R}_{\mathrm{new}}^{\gamma }+{R}_{\mathrm{con}}^{\gamma }$$

Another physics law considered in the model is the conservation of matter. When a new pipe is connected to the bifurcation site, the blood flow in the bifurcation pipe (${Q}_{\mathrm{bif}}$) is conserved as given by Eq. (3):

3 $${Q}_{\mathrm{bif}}{=Q}_{\mathrm{cont}}+{Q}_{\mathrm{term}}$$

From Eq. (3), ${Q}_{\mathrm{cont}}$ and ${Q}_{\mathrm{term}}$ are the blood flows of the continuation pipe and the new pipe, respectively. The addition of the new pipe produces an increase in the blood volume circulating in the bifurcation pipe with a radius ${R}_{\mathrm{bif}}$. To account for this increase, the pressure at the entry of the bifurcation pipe needs to be updated. By rearranging Eq. (1), an expression is obtained for the new pressure at the entry of the bifurcation pipe (${P}_{\mathrm{entry}-\mathrm{end}}$). This new pressure at the bifurcation pipe entry causes that the pressures of all pipes in the path to the root pipe of the tree need to be updated.

4 $${P}_{\mathrm{entry}-\mathrm{end}}={P}_{\mathrm{bif}-\mathrm{end}}+{Q}_{\mathrm{bif}}\frac{8\mu {l}_{\mathrm{bif}}}{\pi {R}_{\mathrm{bif}}^{4}}$$

Our model updates all pressures of the pipes each time a new pipe is connected to the tree while maintaining a predetermined pressure (${P}_{\mathrm{perf}}$) at the entry point of the root segment.

### Main features of liver vasculature

We selected the liver to generate internal vasculature because the liver is a highly vascularized organ that contains at any given time ~ 10% of the total body blood volume in the adult male and adult female (Table 2.14 of ICRP Publication 89 [16]). The liver vascular network, in normal conditions, is very homogenous within the organ volume. Liver human vasculature is unique compared to other human organs as it receives blood from two inlets—the hepatic artery (HA) and hepatic portal vein (HPV)—and drainage occurs via one outlet—the hepatic veins (HV). Oxygenated blood from the HA flows along the HA network and runs analogous to the partially deoxygenated blood from the HPV that circulates through the HPV system. Both inlet systems end at the sinusoids where a mixture of HA and HPV blood occurs. After all metabolic processes take place in the lobules, de-oxygenated blood is extracted from the liver via the HV network, which then passes through the right, middle, or left HV and finally drains into the inferior vena cava.

The anatomy of the liver has been classified using different approaches and patterns [17–20]. In 2000, the Terminology Committee of the International Hepato-Pancreato-Biliary Association presented a universal terminology—the *Brisbane 2000 system*—to avoid confusion and inappropriate use of the terms used to classify the liver [21]. The Brisbane 2000 system adopted the liver segment classification originally proposed by Couinaud [19]. Our work adopts the terminology presented in the Brisbane 2000 system when referring to the segments of the liver.

Another important feature of the liver and its vasculature is that each segment of the liver has its own unique vascular inflow, outflow, and biliary drainage. At each segment, one branch of the HA and one branch of the HPV bring blood to the segment and a branch of the HV drains the blood out of the segment.

In our work, the liver was first divided into segments and branches of the HA, HPV, and HV where each segment embodies reference parameters for vessel radius, length, pressure, and blood flow. To create computational vascular models that take into consideration the independent inflow and outflow that occurs in each liver segment, the ICRP adult mesh-type reference computational phantom (MRCP) livers were utilized [11].

### Development of liver segments and main vasculature

Using the Surgical Anatomy of the Liver application from Emory University^2^ as an illustrative reference and defining a set of cutting planes and surfaces, the ICRP reference livers were divided into eight segments with similar shapes to those visualized in the application. The percentage of total liver volume (PTLV) for each segment was compared against published experimental data. Mise et al. [22] performed 3D reconstruction and volumetric analysis of 107 normal livers from donor candidates and reported values of PTLV for this cohort. The median values of PTLV reported in this study were used as target parameters in the creation of the segments of AFL/AML. Table 1 shows the PTLV values (median and ranges) from Mise et al*.* as well as the values obtained after segmentation was performed in the AFL/AML. Absolute differences between our values and the reference values for the AFL and AML are less than 1.3% and 0.7%, respectively. In both cases, the values listed do not sum to 100%. For the Mise et al*.* data, we report their median values which sum to 99.9% and 97.4% for their male and female study subjects, respectively. In the present study, our percentages sum to 97.4% and 97.3%, respectively, with the additional ~ 3% of liver volume accounting for inter-lobular ligaments and fibrous tissues which spatially define the lobes.

**Table 1:** PTLV values from a reference study reported by Mise et al. [22] and from the segmentation performed in the MRCP AFL/AML (present study)

Liver segments	% of TLV in male liver			Absolute difference	% of TLV in Female Liver			Absolute difference
	Mise et al.		Present study		Mise et al.		Present study	
	Median	Range			Median	Range		
S1	3.9	1.7–8.6	3.5	− 0.40	4.2	1.3–10.1	4.0	− 0.20
S2	7.9	3.0–15.0	8.1	0.20	7.6	2.9–16.1	7.7	0.10
S3	9.8	5.7–19.8	9.5	− 0.30	8.5	4.1–14.6	8.8	0.30
S4	13.4	6.3–20.9	13.2	− 0.20	13.8	5.1–19.9	14.0	0.20
S5	12.3	4.4–20.1	12.1	− 0.20	12.7	4.8–19.4	12.5	− 0.20
S6	7.8	1.4–19.8	7.1	− 0.70	8.0	1.1–20.0	6.7	− 1.30
S7	19.9	7.0–31.3	19.4	− 0.50	16.0	6.0–35.8	17.1	1.10
S8	24.9	11.1–31.9	24.5	− 0.40	26.6	15.6–38.0	26.5	− 0.10

The last step prior the execution of the vessel generation algorithm involves the creation of the main vessels that feed and drain the blood at each liver segment. By using the Emory anatomical model of the liver as a visual guide, and the AFL/AML segments as landmarks, the proper hepatic artery, hepatic portal vein, and a portion of the inferior vena cava including all branches up to the fourth generation were manually constructed using the modeling software Rhinoceros 6.0.^3^ Geometrical parameters including radius and length of each vessel were extracted from Debbaut et al. [23] in which vascular corrosion casting combined with micro-CT imaging and image processing were performed to obtain a detailed description of human liver vasculature. Regarding the hemodynamic parameters, a total hepatic blood flow entering the liver of 100 mL/min per 100 g liver wet weight was considered as suggested by Eipel et al. [24].

According to the anatomical peculiarity of the dual afferent blood supply of the liver, 25% of the total blood entering the liver is oxygenated blood arriving from the proper HA and the other 75% is partially deoxygenated venous blood from the HPV. The pressure value of the proper HA was extracted from Crookston et al. [9]. Normal pressures at the HPV and inferior vena cava were obtained from Lebrec et al. [25].

### Construction of vascular trees and solving intersection of vessel segments

After defining all hemodynamic and geometrical parameters of the constructed main vessels, end pressures and blood flow rates at the main terminal branches that feed and drain each segment were used as inputs for the vessel generation algorithm. With the volume of each liver segment already defined, three terminal branches (from HA, HPV, and HV) of the main vessels were only allowed to end at each liver segment. For each segment, the HA and HPV main terminal branches are used to generate the HA and HPV trees, respectively, while the HV tree was developed starting at the HV main terminal branch.

The developed vessel generation algorithm was utilized to incorporate HA, HPV, and HV trees at each segment of the MRCP AFL/AML. To define the location of the terminal vessels of the trees, random points were homogeneously generated inside each liver segment. The number of generated random points in each segment was selected in a way that the percentage of the number of points in a segment relative to the total number of points in all segments matched the PTLV values shown in Table 1. Using this method, segments with more volume have more terminal points. The total blood flow rate at each liver segment was also assumed to be proportional to the volume of the segment as described by Mise et al. [22]. The blood flow rate in each tree developed was assumed constant for all terminal segments in the tree.

HA, HPV, and HV trees of all nine liver segments, considering that liver segment IV was divided into components IV-A and IV-B, were generated using the algorithm. At each segment (e.g., Segment VII), the endpoint of the HA, HPV, and HV main vessel branch was made to correspond to the locations where the HA, HPV, and HV trees were developed, respectively. At these locations, defined as ‘entry points,’ pressure and blood flow rate are known and defined as ${P}_{\mathrm{perf}}$ and ${Q}_{\mathrm{perf}}$. In our algorithm, the law of conservation of blood flow rate is considered. Thus, at each liver segment and for each type of tree (HA, HPV, or HV), the total blood flow rate is equal to the summation of the blood flow rates at all terminal vessels. As the terminal blood flow rate is the same for all terminal vessels, ${Q}_{\mathrm{perf}}$ can be represented as

5 $${Q}_{\mathrm{perf}}{=\sum_{i=1}^{{N}_{\mathrm{term}}}{Q}_{i}={N}_{\mathrm{term}}\cdot {Q}_{\mathrm{term}}}$$

In Eq. (5), ${N}_{\mathrm{term}}$ is the number of terminal points in a segment and ${Q}_{\mathrm{term}}$ is the terminal blood flow rate that was defined earlier as constant for all terminal vessels in the tree. In each segment, the same terminal points are used to generate the three types of virtual trees. In this way, HA, HPV, and HV trees are connected at their terminal vessels allowing for closed circulation of blood inside the segment similar to what occurs in real human liver vasculature.

In real vascular trees, one vessel does not intersect with any other vessels in the same network except from the vessel where it emerges and the vessel branches that originate from itself. During the process of vessel generation in our model, a constraint of non-intersecting vessels was incorporated into the algorithm and referred to as ‘*Self-Intersection*’ restriction. At each iteration of the algorithm in which several candidate new vessel pipes are constructed, the self-intersection restriction was applied to avoid intersection between a selected new pipe and another vessel of the tree at a location different from the bifurcation site. At any iteration, if the shortest candidate new pipe is not free of intersections, the following shortest pipe is checked and the shortest non-self-intersecting pipe is selected as the permanent connection to the tree.

During the development of vascular networks, the volume to perfuse is the same when creating arterial and venous trees. Assuming two different trees are generated in the same volume, one for the arterial and one for the venous circulation, it is important to avoid the creation of venous pipes that could intersect arterial pipes except for the terminal pipes from each tree that end at the same terminal point. Thus, two types of intersections could occur during the generation of a tree when another tree was previously created in the same volume. The intersection of a terminal vessel of a tree with a terminal of vessel of another tree that shares the same terminal point is defined as ‘*acceptable intersection*.’ Acceptable intersections do not need to be avoided as they are needed to create a closed vascular loop. Intersections of vessels of different trees that do not share the same terminal points are defined as ‘*unacceptable intersections*’ and need to be avoided during vessel generation. A function that detects intersections was incorporated inside the algorithm to check if each vessel created overlaps a vessel from another tree. If there is at least one ‘*unacceptable interception*’ with the new candidate pipe, the new pipe is rejected and the next candidate pipe with the shortest distance to the existing tree is evaluated. Once a new pipe free of unacceptable intersection is found, it is added to the tree as a permanent connection.

To accommodate the blood flow when a new permanent pipe is added to the tree, all radii of bifurcation pipes that are in the path from the new permanent pipe to the root segment increase as defined by Murray’s law. These growths are essential in the algorithm and allow the development of the tree at each iteration. Due to these growths during the tree development, it is possible that one of the bifurcation pipes in the tree increases in radius sufficiently enough to potentially produce an intersection between the bifurcation pipe and another pipe of a previously constructed tree. Although this event is not frequent, it is more probable to occur in regions with less free space (e.g., near the root segments of each tree). To minimize or eliminate the number of these unacceptable intersections, the other trees generated in the same segment were modified by scaling their pipe’s radii by a factor of two. This artificial tree, with twice the original radii of the pipes, was used to check for intersections each time a new vessel was created in the tree under construction. With this solution in place, the number of unacceptable intersections caused by the natural growth of the tree was eliminated or drastically reduced in all trees developed.

At each of the nine segments of the reference liver, HA, HPV, and HV trees were created from the three entry point locations defined by the branches of the main vessels constructed. In total, 27 vascular trees were generated in the AML and AFL independently. About 6000 total blood vessels were created in the vascular networks of both the adult male and adult female liver.

### Tetrahedralization of vascular liver models

The minimum vessel radius modeled is approximately 0.1 mm. To incorporate such details in Monte Carlo (MC) radiation transport simulations, tetrahedral mesh-type format was selected [26]. In addition, the computation speed using MC transport code PHITS [27] for calculations of dose coefficients in the adult MRCP [28] has been reported to be faster in that mesh geometry than using the original voxelized phantom geometry from ICRP Publication 110 [6].

**Fig. 3:**
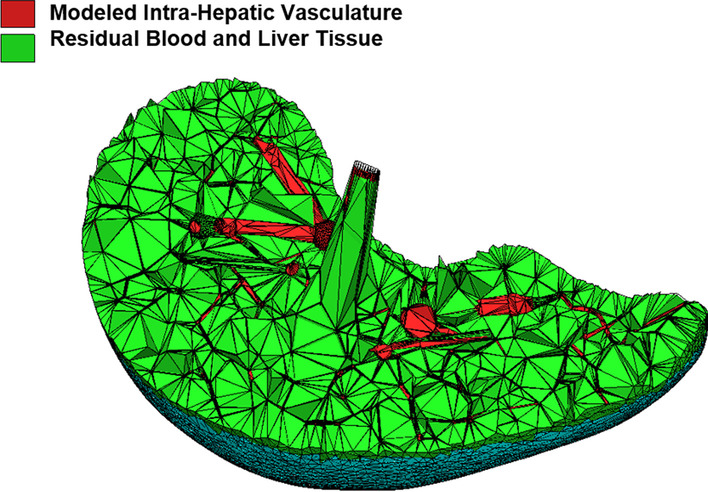
Tetrahedral mesh-type model of the AML. Red tetrahedrons represent the vascular model generated, and green tetrahedrons are a homogenous mixture of residual blood and liver tissue

The AML and AFL with the detailed vascular networks were exported from *Rhinoceros* 6.0 in OBJ format using different organ tag numbers for the vascular models. The tetrahedralization process was performed using the POLY2TET software [29]. The tetrahedral models of vascularized AML and AFL have about 1.4 and 0.9 million tetrahedrons, respectively. Visualization of the tetrahedral AML model with internal virtual vasculature using TETVIEW is shown in Fig. 3.

### Application in radiopharmaceutical therapy: calculation of radionuclide S values

Organ absorbed dose in radiopharmaceutical dosimetry under the MIRD schema is computed as the product of the time-integrated activity $\tilde{A }\left({r}_{S}\right)$, assessed via quantitative imaging, and the radionuclide *S* value [30]. Assuming radiation emissions from a source region ${r}_{S}$, the absorbed dose $D\left({r}_{\mathrm{T}}\right)$ to a target region ${r}_{\mathrm{T}}$ is calculated as

6 $$D\left({r}_{\mathrm{T}}\right)=\sum_{{r}_{s}}\tilde{A }\left({r}_{S}\right)S({r}_{\mathrm{T}}\leftarrow {r}_{S})$$

where $\tilde{A }\left({r}_{S}\right)$ is the number of nuclear decays within the source region. $S\left({r}_{\mathrm{T}}\leftarrow {r}_{S}\right)$ is the mean absorbed dose to the target region per nuclei decay in the source region and is computed using the following expression:

7 $$S\left({r}_{\mathrm{T}}\leftarrow {r}_{S}\right)=\sum_{i}{E}_{i}{Y}_{i}\Phi ({r}_{\mathrm{T}}\leftarrow {r}_{S}, {E}_{i})$$

where ${E}_{i}$ and ${Y}_{i}$ are the energy and yields of the *i*th nuclear transformation of the radionuclide, respectively, and $\Phi \left({r}_{\mathrm{T}}\leftarrow {r}_{S},{E}_{i}\right)$ is the specific absorbed fraction (SAF) for a radionuclide particle of energy ${E}_{i}$ for a given source–target combination. The SAF is defined as the ratio of absorbed fraction (AF—the fraction of the particle energy emitted within the source region that is deposited in the target region) and the target mass:

8 $$\mathrm{SAF}=\Phi \left({r}_{\mathrm{T}}\leftarrow {r}_{S}, {E}_{i}\right)=\frac{\mathrm{AF}\left({r}_{T} \leftarrow { r}_{S}, { E}_{i}\right)}{m({r}_{\mathrm{T}})}$$

SAF and *S* values calculations rely on anatomical patient models in which organs are modeled as single-region volumes where organ parenchyma and blood are homogenously combined. Using this single-region model for the liver, and considering liver (L) as a homogeneous mixture of liver parenchyma (LP) and liver blood (LB) with mass ${m}_{\mathrm{L}}$, for a specific monoenergetic particle, SAF value is calculated as

9 $$\mathrm{SAF} \left(\mathrm{L}\leftarrow \mathrm{L}\right) = \frac{\mathrm{AF} \left(\mathrm{L}\leftarrow \mathrm{L}\right)}{{m}_{\mathrm{L}}}$$

Under the single-region model, $\mathrm{SAF} \left(\mathrm{L}\leftarrow \mathrm{L}\right)$ is the approximation used for the ideal case in which LP is both source and target—$\mathrm{SAF} \left(\mathrm{LP}\leftarrow \mathrm{LP}\right)$—and for the case in which LB is the source and LP is the target region—$\mathrm{SAF} \left(\mathrm{LP}\leftarrow \mathrm{LB}\right)$. These approximations are due to the lack of an internal vasculature model in which liver blood content is differentiated from the liver parenchyma.

With the liver vascular model presented in this study, it is possible to have more refined approximations for both $\mathrm{SAF} \left(\mathrm{LP}\leftarrow \mathrm{LP}\right)$ and $\mathrm{SAF} \left(\mathrm{LP}\leftarrow \mathrm{LB}\right)$. Considering this aspect, a dual-region liver model is proposed. The first region is the *liver inside blood vessels* (LIBV) referring to the vascular model created inside the liver. The other one is *liver outside blood vessels* (LOBV) defined as a homogeneous mixture of the residual blood not modeled (any vessel below 0.1 mm in radius including capillaries and blood sinuses) and the liver parenchymal tissue. Although the vascular networks created for the AFL/AML do not account for the total blood content in the adult reference livers, having some fraction of the blood model would have an impact on internal dosimetry. We hypothesize that some difference from the single-region approximations would be expected for short-range particles that have the chance to deposit their energy completely inside the vascular network (blood self-dose). Using the dual-region liver model proposed, $\mathrm{SAF} \left(\mathrm{LP}\leftarrow \mathrm{LP}\right)$ can be approximated:

10 $$\mathrm{SAF}\left(\mathrm{LP}\leftarrow \mathrm{LP}\right) \approx \mathrm{SAF}\left(\mathrm{LOBV}\leftarrow \mathrm{LOBV}\right)= \frac{\mathrm{AF}\left(\mathrm{LOBV}\leftarrow \mathrm{LOBV}\right)}{{m}_{\mathrm{LOBV}}}$$

Under the assumption of a dual-region liver model, $\mathrm{SAF} \left(\mathrm{LP}\leftarrow \mathrm{LB}\right)$ can be obtained as

11 $$\mathrm{SAF}\left(\mathrm{LP}\leftarrow \mathrm{LB}\right) \approx \mathrm{SAF}\left(\mathrm{LOBV}\leftarrow \mathrm{LB}\right)= \frac{{f}_{\mathrm{BV}} \cdot \mathrm{AF}\left(\mathrm{LOBV}\leftarrow \mathrm{LIBV}\right)+ \left(1-{f}_{\mathrm{BV}}\right) \cdot \mathrm{AF}\left(\mathrm{LOBV}\leftarrow \mathrm{LOBV}\right) }{{m}_{\mathrm{LOBV}}}$$

where ${f}_{\mathrm{BV}}$ is the fraction of total liver blood mass explicitly modeled within the blood vasculature created.

As previously mentioned, the sinusoids store the mixture of blood from the hepatic arterial and portal circulation. In addition, 60% of blood content in the liver is stored in the sinusoids, while the other 40% of blood is contained in main vessels, pre-capillaries, and capillaries. As our virtual vasculature does not model any vessel below 0.1 mm radius, the vasculature models account for only 13% and 15% of total blood volume content in the AML and AFL, respectively. We hypothesize, however, that while these percentages are small, it is in these major blood vessels that the greatest impact on macroscale dosimetry will be seen via explicit accounting for radionuclide blood self-dose.

MC simulations to compute the AF values were performed with the PHITS transport code v3.24 [27] using the University of Florida HiPerGator computing cluster. Monoenergetic alpha particles, electrons,^4^ and photons were defined as sources in the single-region liver model and independently in both regions (LIBV and LOBV) of the dual-region liver model. In the single-region liver model, particle sources were randomly sampled within the homogenized mixture of liver blood and liver parenchyma. In the dual-region liver model, two separate simulations were performed in which the particle sources were uniformly distributed in either the LIBV and LOBV regions, respectively. A total of 24 alpha particle energies were sampled from 0.5 to 12 MeV in increments of 0.5 MeV along a linear scale. For electrons and photons, a logarithmic energy grid from 10 keV to 10 MeV was used (26 energies in total for each). Particle histories were generated giving relative errors in energy deposition tallies below 1% for both single and dual regions. Table 2 provides the details of the MC simulations performed. Table 3 provides the details on the elemental compositions and mass densities assumed in both the single-region and dual-region models of reference adult female and reference adult male to include: liver parenchyma (LP), liver blood (LB as well as LIBV), liver tissue regions outside the modeled blood vessels (LOBV), and the fully homogenized liver of the single-region model (L).

**Table 2:** Details of the PHITS transport computations and data post-processing

Item	Description	References
Code and version	PHITS v3.24	[27]
Source description	s-type = 24. Particles are produced uniformly from each tetrahedron, which belong to the specified universe	[27]
Cross sections	PDL97 for photons	[33]
	EGS5 for photons, electrons, and positrons	[34]
	INCL for nucleons and light ions	[35]
Transport parameters	Secondary electrons were followed for photon simulations. Alpha particles were simulated down to 0.1 MeV/nucleon, while gammas, electrons, and positrons were simulated down to 1 keV	[27]
Variance reduction	No variance reduction techniques were utilized for this study	
Statistical uncertainties and history numbers	For single-region liver model: 1 million photons, electrons, positrons, and alpha particles histories were simulated independently at each energy, relative errors in energy deposition tallies were below 1%	[27]
	For dual-region liver model: 1 million photons, electrons, positrons, and alpha particles histories were simulated independently at each energy, relative errors in energy deposition tallies were below 1% except for 10 keV electrons and positrons and 0.5 MeV alpha particles in which 10 million particles histories were simulated to achieve relative errors in energy deposition tallies below 1%	
Data and post-processing	Energy deposited (MeV/source) was tallied in the single-region liver model. Absorbed fractions were calculated by normalizing the results to the particle source energy. Energy deposited (MeV/source) was tallied in the LOBV region of the dual-region liver model. Absorbed fractions from the following source–target combinations: (LOBV ← LOBV) and (LOBV ← LIBV) were calculated by normalizing the results to the particle source energy at each target. The fraction of blood mass was used to weight-average the absorbed fractions and normalizing by the mass of the target region (LOBV) (See Eq. 11)	[27]

**Table 3:** Tissue elemental compositions, mass densities, and total tissue masses assigned in the PHITS transport models

	Elemental composition (% by mass)										Density (g cm^3^)	Source	
Tissue component	H	C	N	O	Na	P	S	Cl	K	Fe			
Adult healthy liver	10.20	13.90	3.00	71.60	0.20	0.30	0.30	0.20	0.30	0.00	1.06	ICRU 46	
Adult whole blood	10.20	11.00	3.30	74.50	0.10	0.10	0.20	0.30	0.20	0.10	1.06	ICRU 46	
*Adult female liver (AFL)*													*Total mass (kg)*
Homogeneous liver (L)	10.20	13.24	3.07	72.26	0.18	0.25	0.28	0.22	0.28	0.02	1.06		1.81
Liver inside BV (LIBV)	10.20	11.00	3.30	74.50	0.10	0.10	0.20	0.30	0.20	0.10	1.06	ICRU 46	0.06
Liver outside BV (LOBV)	10.20	13.32	3.06	72.18	0.18	0.26	0.28	0.22	0.28	0.02	1.06		1.75
*Adult male liver (AML)*													*Total mass (kg)*
Homogeneous liver (L)	10.20	13.21	3.07	72.29	0.18	0.25	0.28	0.22	0.28	0.02	1.06		2.36
Liver inside BV (LIBV)	10.20	11.00	3.30	74.50	0.10	0.10	0.20	0.30	0.20	0.10	1.06	ICRU 46	0.07
Liver outside BV (LOBV)	10.20	13.28	3.06	72.22	0.18	0.26	0.28	0.22	0.28	0.02	1.06		2.28

Radionuclide decay data on emission energies and yields were taken from the MIRD Monograph on Radionuclide Data and Decay Schemes as given in the MIRD-07.RAD and MIRD-07.BET data files [31]. The full energy spectra for both beta particles and positrons were considered in lieu of considering only their mean energies. All computed *S* values were performed using a Python script with SAF interpolation through particle energies using piecewise cubic Hermite interpolation polynomials (PCHIP). *S* values were computed for five different radiation classes: (1) photons, (2) beta particles, (3) electrons, (4) alpha particles, and (5) alpha recoil particles. For the last class, the SAF values were interpolated at 2 MeV alpha particle, an approach previously adopted by the ICRP Publication 133 [32]. *S* values for members of alpha-emitter decay chains were computed and reported independently for the parent radionuclide and all individual progeny.

## Results

### Liver vasculature model

**Fig. 4:**
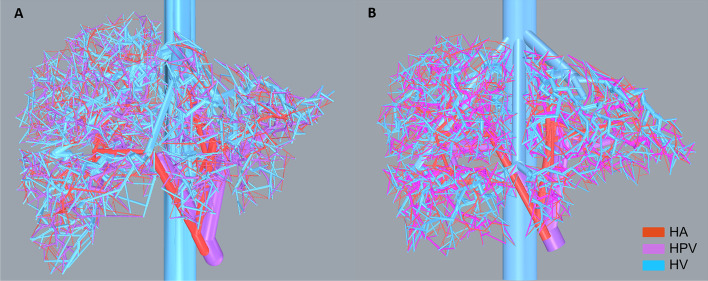
Main vessels and vascular trees generated inside the **A** AML and the **B** AFL

Figure 4 shows the final vascular models for the AML and AFL. HA, HPV, and HV vasculatures are displayed in different colors as shown in the legend. Any self-intersections, unacceptable intersections, and intersections with the outer surface mesh of the liver were eliminated in the final models.

**Fig. 5:**
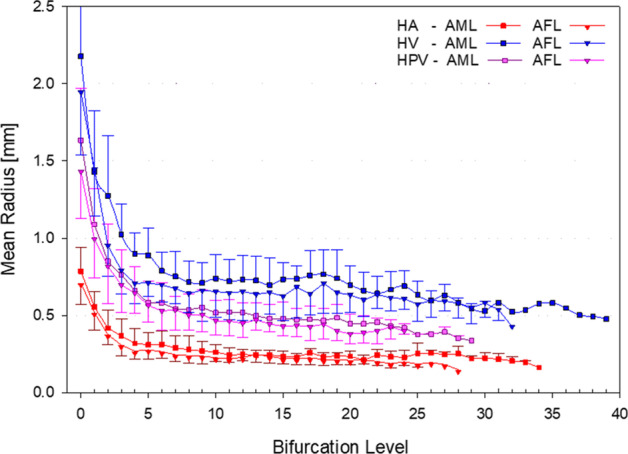
Mean vessel radius per bifurcation level for each type of virtual vasculature created in the AML and AFL. Bars are the standard deviation associated with each mean value

To characterize virtual vascular trees and compare them with other models, two parameters are usually referenced: the bifurcation level and the Strahler order. The former is defined as the number of proximal bifurcations from a specific vessel along its path to the root vessel. In the developed models, vessels have been classified depending on the bifurcation level and grouped accordingly. The mean radius of vessels with equal bifurcation level is calculated for each type of vasculature (HA, HPV, and HV) in each of our liver models (AML and AFL). The mean radius with its associated standard deviation at each bifurcation level is shown in Fig. 5. Square and down-triangle symbols were used for the mean radii of the AML and AFL, respectively. Different colors were used to differentiate between the HA, HPV, and HV vascular trees.

**Fig. 6:**
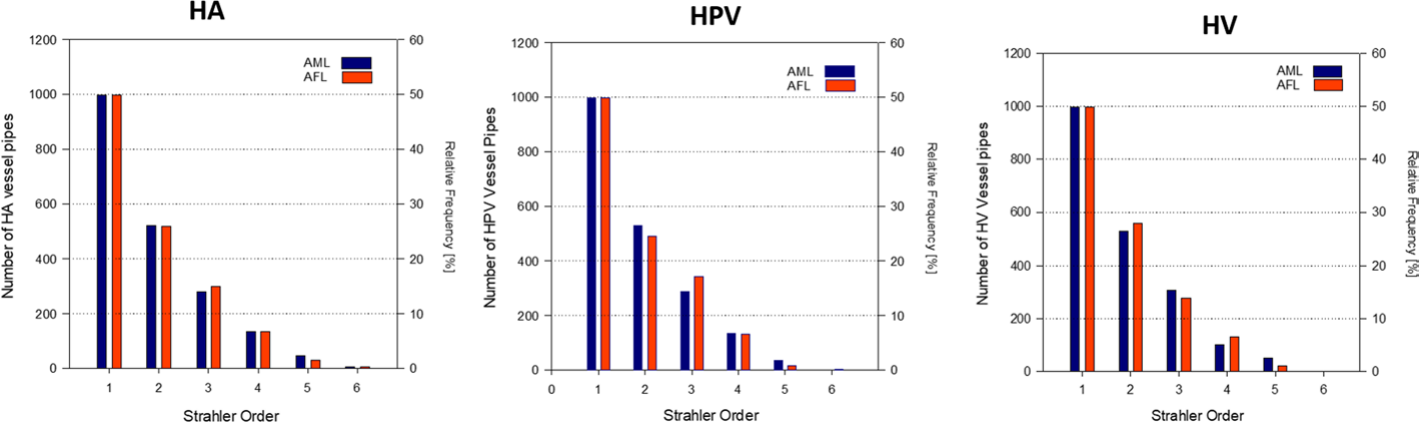
Distribution of virtual vessels of HA, HPV, and HV vascular trees per Strahler order in AML/AFL

The Strahler order is utilized to reflect the morphometry of the developed trees. A Strahler order of one was assigned to all terminal vessels. If a bifurcation vessel has two daughter vessels with the same Strahler order, the Strahler order of the daughters plus one is assigned to the bifurcation vessel. If the daughter's vessels have different Strahler orders, the highest of the Strahler order of the daughters will be assigned to the bifurcation vessel. Figure 6 shows the distribution of vessels per Strahler order for hepatic arterial, hepatic portal, and hepatic venous virtual trees in both AML and AFL models.

### Liver dosimetry

Specific absorbed fractions (SAF) assuming the single-region and the dual-region liver models were calculated using Eq. (9) and Eqs. (10) and (11), respectively. In the proposed dual-region liver model, blood decays were modeled in two stages: (1) sites within explicitly modeled hepatic vessels (LIBV), and (2) residual blood not modeled and liver parenchyma (LOBV).

**Fig. 7:**
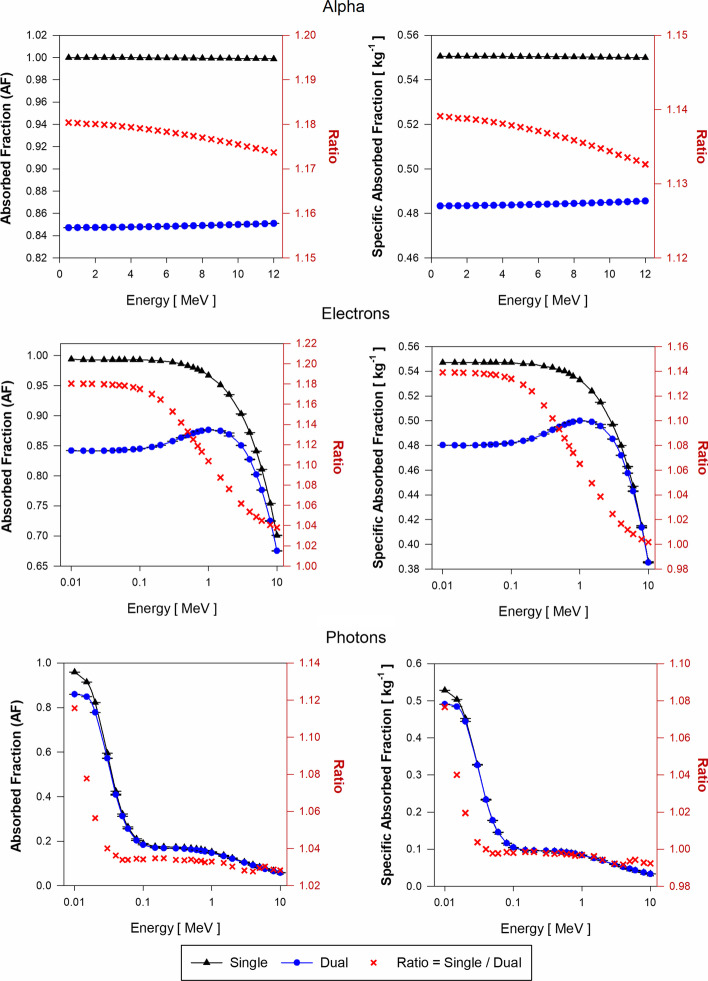
Approximations of $\mathrm{AF}\left(\mathrm{LP}\leftarrow \mathrm{LB}\right)$ and $\mathrm{SAF}\left(\mathrm{LP}\leftarrow \mathrm{LB}\right)$ for monoenergetic alpha particles (Top), electrons (Center), and photons (Bottom) using the single-region liver model (black triangles) and the dual-region liver model (blue circles) in tetrahedral mesh-type format for the reference adult female liver (AFL)

**Fig. 8:**
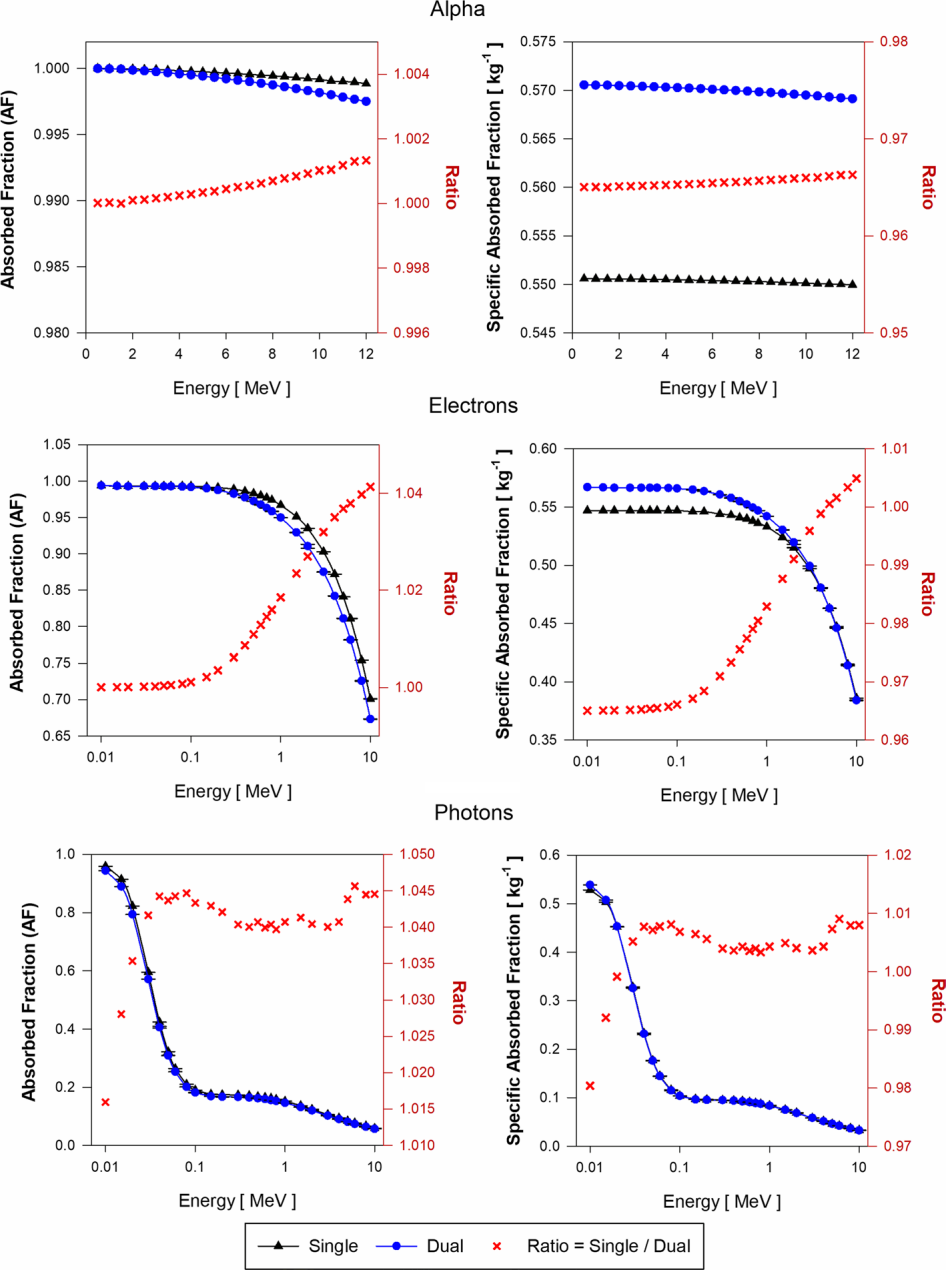
Approximations of $\mathrm{AF}\left(\mathrm{LP}\leftarrow \mathrm{LP}\right)$ and $\mathrm{SAF}\left(\mathrm{LP}\leftarrow \mathrm{LP}\right)$ for monoenergetic alpha particles (Top), electrons (Center), and photons (Bottom) using the single-region liver model (black triangles) and the dual-region liver model (blue circles) in tetrahedral mesh-type format for the reference adult female liver (AFL)

**Fig. 9:**
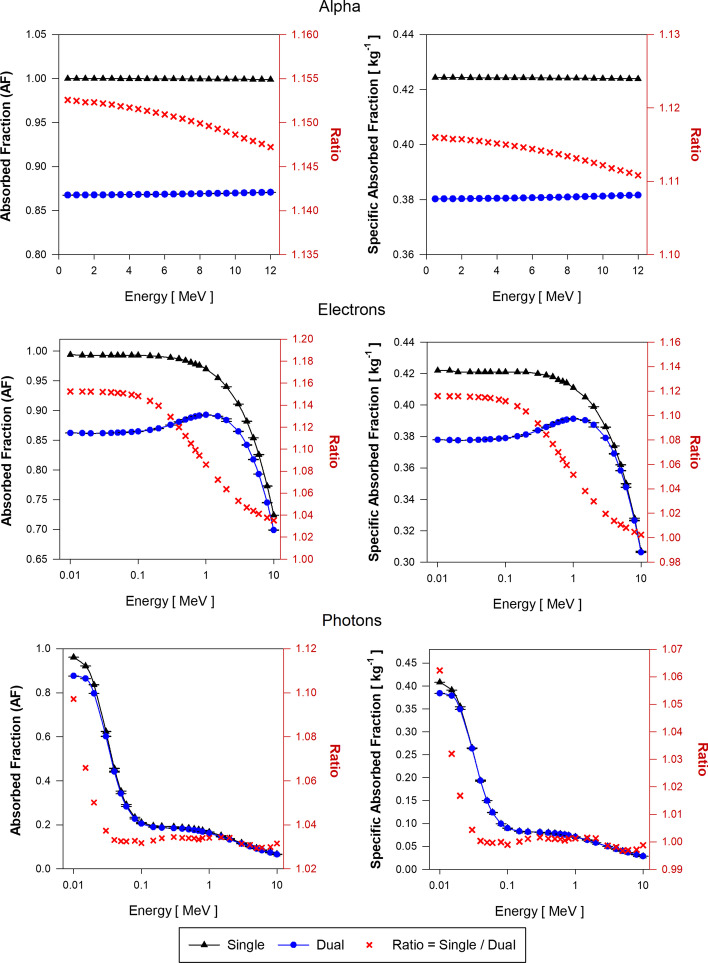
Approximations of $\mathrm{AF}\left(\mathrm{LP}\leftarrow \mathrm{LB}\right)$ and $\mathrm{SAF}\left(\mathrm{LP}\leftarrow \mathrm{LB}\right)$ for monoenergetic alpha particles (Top), electrons (Center), and photons (Bottom) using the single-region liver model (black triangles) and the dual-region liver model (blue circles) in tetrahedral mesh-type format for the reference adult male liver (AML)

**Fig. 10:**
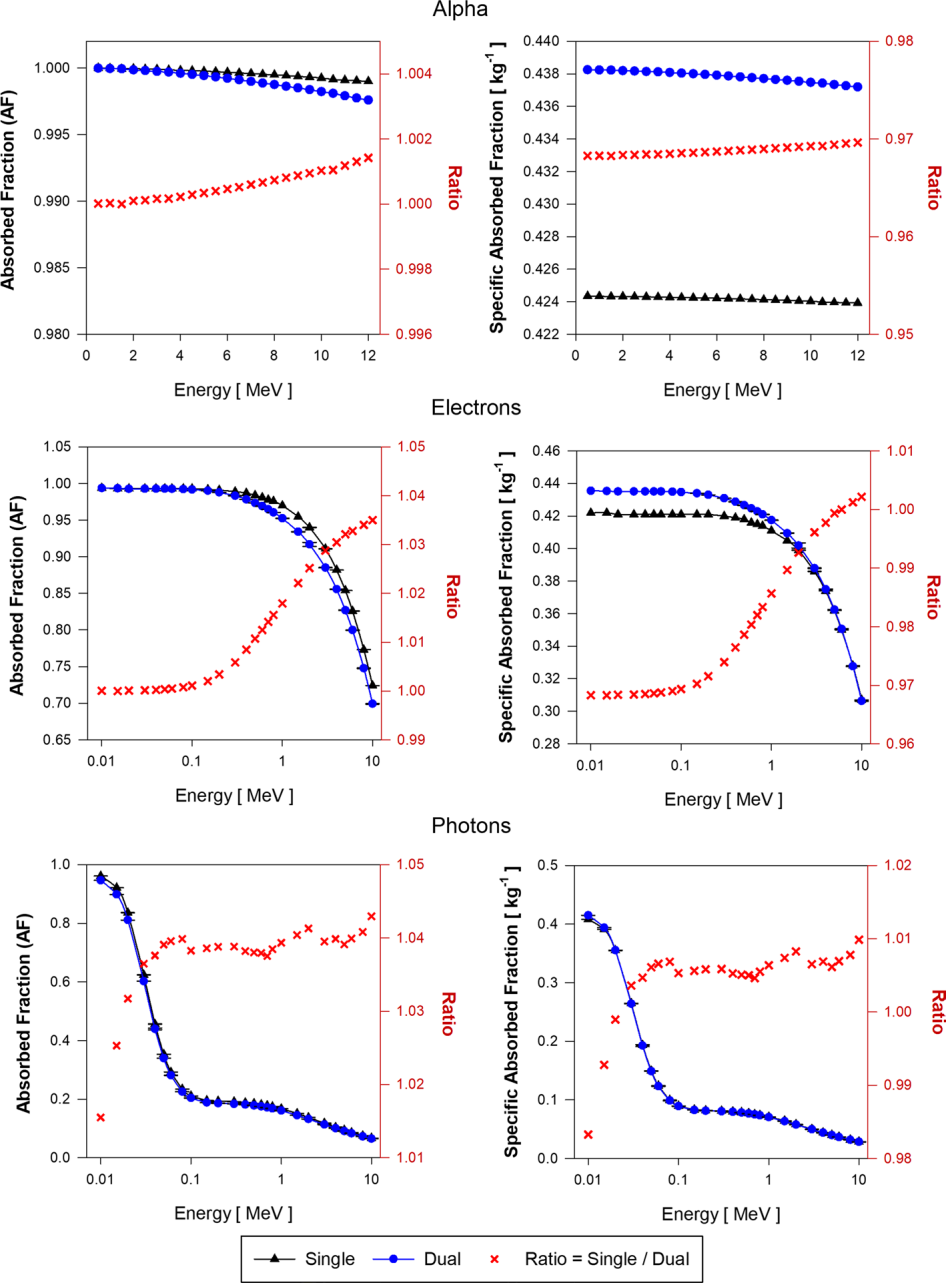
Approximations of $\mathrm{AF}\left(\mathrm{LP}\leftarrow \mathrm{LP}\right)$ and $\mathrm{SAF}\left(\mathrm{LP}\leftarrow \mathrm{LP}\right)$ for monoenergetic alpha particles (Top), electrons (Center), and photons (Bottom) using the single-region liver model (black triangles) and the dual-region liver model (blue circles) in tetrahedral mesh-type format for the reference adult male liver (AML)

Figure 7 shows the AF and SAF values for liver blood (LB) sources of alpha particles, electrons, and photons sources in both the single-region and dual-region tetrahedral mesh models of the adult female liver (AFL). Each plot also gives the ratio of values from the single-region and dual-region models. Figure 8 shows similar data but for decay sites in liver parenchyma (LP) for the AFL. Corresponding data for the AML are shown in Figs. 9 and 10, respectively.

## Discussion

### Morphometric analysis of the vascular models in the AFL/AML

As shown in Fig. 5, the mean radius decreases as the bifurcation level increases in all types of trees for the AFL and AML models. Mean radii from all trees created in AML are higher than the mean radii of the AFL vascular trees. The difference is caused by a higher total blood flow in the AML compared to that in the AFL model. Due to the high variability of liver vasculature between individuals [36], a morphometric basis comparison of our results with real or modeled human vasculature is difficult to perform. Nevertheless, similar decreasing trends have been reported for the mean radii of virtual and real vascular trees in the liver and other human organs [8, 23, 37].

Figure 6 shows the number of vessels $(N)$ created per Strahler order $(\mathrm{SO})$ for the HA, HPV, and HV trees in both AML and AFL models. The distributions of vessels for the AML and AFL vascular trees are very similar and exhibit exponential decay trends with the increase in the Strahler order. The data in Fig. 6 were fit using an exponential decay function $N=a\cdot{e}^{-b\cdot\mathrm{SO}}$. The coefficients of determination $({R}^{2})$ were greater than 0.992 in all fits. Similar exponential decay trends have been reported in other computational vascular trees [38, 39].

### AF and SAF values from the single- and dual-region models of the AFL/AML

Figure 7 displays the approximations of $\mathrm{AF}\left(\mathrm{LP}\leftarrow \mathrm{LB}\right)$ and $\mathrm{SAF}\left(\mathrm{LP}\leftarrow \mathrm{LB}\right)$ for the AFL model. For alpha particles, the current single-region liver model is shown to give a value of $\mathrm{AF}\left(\mathrm{LP}\leftarrow \mathrm{LB}\right)$ that is between 17 and 18% higher than given by the dual-region liver model where blood self-dose is considered within its modeled vessels. Correspondingly, values of $\mathrm{SAF}\left(\mathrm{LP}\leftarrow \mathrm{LB}\right)$ in the single-region model are between 13 and 14% higher than given by the dual-region liver model. The SAF values are obtained as the quotient of the AF and the target mass. The mass of the single-region AFL model (${m}_{\mathrm{L}})$ is 3.5% higher than the target mass (${m}_{\mathrm{LOBV}})$ in the dual-region AFL model. Thus, changes in target mass only partially account for SAF percent differences.

For low-energy electrons (less than 100 keV) in the AFL, $\mathrm{AF}\left(\mathrm{LP}\leftarrow \mathrm{LB}\right)$ calculated using the single-region model are larger by ~ 18% as compared to those obtained with the dual-region liver model. Above 100 keV and up to 10 MeV, the ratio of $\mathrm{AF}\left(\mathrm{LP}\leftarrow \mathrm{LB}\right)$ values in the single-region to values in the dual-region models decreases from 1.18 to 1.04. $\mathrm{SAF}\left(\mathrm{LP}\leftarrow \mathrm{LB}\right)$ values using the single-region liver model are up to 14% higher than those of the dual-region liver model for electrons below 100 keV. In the dual-region liver model, very low-energy electrons generated in the LIBV region deposit all the energy locally and never reach the LOBV region. The occurrence of similar events is impossible to discern in the single-region liver model as there is no differentiation between liver tissue and liver blood. At higher electron energies, the probability of these events decreases due to the increase in electron range in relation to the size of the blood vessel structures modeled (LIBV region). Moreover, with the increase in electron energy, Bremsstrahlung radiation emissions increase, resulting in a further decline in local blood self-dose.

Values of $\mathrm{AF}\left(\mathrm{LP}\leftarrow \mathrm{LB}\right)$ obtained during photon transport using the single-region model are up to 12% greater than the $\mathrm{AF}\left(\mathrm{LP}\leftarrow \mathrm{LB}\right)$ values using the dual-region at low photon energies (about 10 keV). The ratio of $\mathrm{AF}\left(\mathrm{LP}\leftarrow \mathrm{LB}\right)$ values in the two liver models decreases drastically between 10 and 40 keV and remains about 1.03 for photon energies between 50 keV and 10 MeV. The photon $\mathrm{SAF}\left(\mathrm{LP}\leftarrow \mathrm{LB}\right)$ values obtained using both models are very similar for all energies except for below 40 keV which yields an SAF ratio increase up to $\sim$ 1.08.

Figure 8 displays the approximations of $\mathrm{AF}\left(\mathrm{LP}\leftarrow \mathrm{LP}\right)$ and $\mathrm{SAF}\left(\mathrm{LP}\leftarrow \mathrm{LP}\right)$ for the AFL model. For alpha particles, the energy depositions are similar for both the single- and dual-region models and the AF ratio is about 1.0 at all energies. For alpha $\mathrm{SAF}\left(\mathrm{LP}\leftarrow \mathrm{LP}\right)$ values, the single-region liver model is 3.5% higher than the $\mathrm{SAF}\left(\mathrm{LP}\leftarrow \mathrm{LP}\right)$ calculated using the dual-region model. As $\mathrm{AF}\left(\mathrm{LP}\leftarrow \mathrm{LP}\right)$ values using both models are almost identical, the SAF ratio is $\sim$ 3.5%, which is explained by the 3.5% excess in the mass of the single-region liver model compared to the LOBV region mass in the dual-region liver model.

For low-energy electrons, $\mathrm{AF}\left(\mathrm{LP}\leftarrow \mathrm{LP}\right)$ values using both models are identical and the AF ratio increases up to 1.04 for 10 MeV electrons. Assuming equal AF values for both models, and about 3.5% excess in the mass of the single-region model compared to the dual-region model, $\mathrm{SAF}\left(\mathrm{LP}\leftarrow \mathrm{LP}\right)$ values using the single-region model are lower by 3.5%. As electron energy increases above 0.1 MeV, the lower values of $\mathrm{SAF}\left(\mathrm{LP}\leftarrow \mathrm{LP}\right)$ using the single-region model are compensated by their higher values (up to 4%) in the single-region model in the AF calculations.

For low-energy photon sources, the ratio of $\mathrm{AF}\left(\mathrm{LP}\leftarrow \mathrm{LP}\right)$ using the single-region model to that in the dual-region model increases from 1.01 to 1.04. Above 30 keV, the single-region model is shown to give higher $\mathrm{AF}\left(\mathrm{LP}\leftarrow \mathrm{LP}\right)$ values by ~ 4%. For photons above 30 keV, the 3.5% mass excess of the single-region model compensates for this 4% increase in $\mathrm{AF}\left(\mathrm{LP}\leftarrow \mathrm{LP}\right)$, thus giving a $\mathrm{SAF}\left(\mathrm{LP}\leftarrow \mathrm{LP}\right)$ ratio of nearly 1.0.

Approximations of AF and SAF for the two target–source combinations ($\mathrm{LP}\leftarrow \mathrm{LB}$ and $\mathrm{LP}\leftarrow \mathrm{LP}$) using the AML single-region and dual-region models are shown in Figs. 9 and 10, respectively. For all alpha particles and low-energy electrons, values of $\mathrm{AF}(\mathrm{L}\leftarrow \mathrm{L})$ and $\mathrm{SAF}(\mathrm{L}\leftarrow \mathrm{L})$ are shown to be higher by 15% and 11.5%, respectively, as compared to values given by the dual-region liver model, specifically $\mathrm{AF}(\mathrm{LOBV}\leftarrow \mathrm{LB})$ and $\mathrm{SAF}(\mathrm{LOBV}\leftarrow \mathrm{LB})$. For photons above 30 keV, AF ratios are $\sim$ 1.04 and SAF ratios are $\sim$ 1. Below 30 keV, ratios of AF and SAF for photons increase up to 1.10 and 1.06, respectively. For the $\mathrm{LP}\leftarrow \mathrm{LP}$ source/target combination, similar tendencies were found in both single-region and dual-region adult male liver models as compared to those seen in the adult female liver models.

### Computed S values for alpha, beta, and Auger emitter radionuclides

*S* values were computed for 22 radionuclides with application to radiopharmaceutical therapy, along with 10 radionuclides commonly used in diagnostic imaging. For the alpha emitters, *S* values were also computed for some 14 different decay chain progeny corresponding to six different parent alpha emitters. For both the parent and progeny radionuclides, the reported *S* values are for individual radionuclides and thus branching ratios and biokinetics-derived sums must be applied to compute a total *S* value for the relevant alpha-emitter decay series. Tables 4 and 5 provide these *S* values for adult female liver, while Tables 6 and 7 provide them for the adult male liver.

**Table 4:** Approximations of $\mathrm{S}\left(\mathrm{LP}\leftarrow \mathrm{LB}\right)$ and $\mathrm{S}\left(\mathrm{LP}\leftarrow \mathrm{LP}\right)$ values for 22 radionuclides (and 14 additional alpha-emitter decay progeny) with applications to radiopharmaceutical therapy using the single-region and dual-region tetrahedral mesh models of the reference adult female liver (AFL)

Radionuclide	*S values (mGy/MBq-s)*					
	Approximations to S(LP ← LB)		Ratio	Approximations to S(LP ← LP)		Ratio
	S(L ← L)	S(LOBV ← LB)		S(L ← L)	S(LOBV ← LOBV)	
Alpha emitters*						
At-211	2.22E−04	1.95E−04	1.137	2.22E−04	2.30E−04	0.966
(Po-211)	6.69E−04	5.89E−04	1.136	6.69E−04	6.93E−04	0.966
(Bi-207)	3.18E−05	3.11E−05	1.022	3.18E−05	3.19E−05	0.997
Bi-212	2.40E−04	2.14E−04	1.124	2.40E−04	2.48E−04	0.969
(Po-212)	7.90E−04	6.96E−04	1.135	7.90E−04	8.18E−04	0.966
(Tl-208)	8.96E−05	8.60E−05	1.042	8.96E−05	9.05E−05	0.989
Bi-213	5.07E−05	4.64E−05	1.094	5.07E−05	5.20E−05	0.976
(Po-213)	7.53E−04	6.63E−04	1.136	7.53E−04	7.80E−04	0.966
(Tl-209)	8.69E−05	8.26E−05	1.051	8.69E−05	8.77E−05	0.990
(Pb-209)	1.73E−05	1.55E−05	1.114	1.73E−05	1.78E−05	0.971
Ra-223	5.18E−04	4.56E−04	1.136	5.18E−04	5.37E−04	0.966
(Rn-219)	6.08E−04	5.35E−04	1.136	6.08E−04	6.30E−04	0.966
(Po-215)	6.64E−04	5.84E−04	1.136	6.64E−04	6.87E−04	0.966
(Pb-211)	4.02E−05	3.71E−05	1.084	4.02E−05	4.12E−05	0.978
(Bi-211)	5.90E−04	5.19E−04	1.137	5.90E−04	6.11E−04	0.966
(Tl-207)	4.28E−05	3.95E−05	1.084	4.28E−05	4.38E−05	0.978
(Po-211)	6.69E−04	5.89E−04	1.136	6.69E−04	6.93E−04	0.966
Ac-225	5.22E−04	4.59E−04	1.137	5.22E−04	5.41E−04	0.965
(Fr-221)	5.67E−04	4.99E−04	1.137	5.67E−04	5.88E−04	0.966
(At-217)	6.35E−04	5.59E−04	1.137	6.35E−04	6.58E−04	0.966
Th-227	5.37E−04	4.73E−04	1.136	5.37E−04	5.56E−04	0.966
*Beta and positron emitters*						
Sr-89	5.03E−05	4.67E−05	1.079	5.03E−05	5.14E−05	0.980
Y-90	7.93E−05	7.46E−05	1.063	7.93E−05	8.04E−05	0.986
I-124	3.24E−05	3.13E−05	1.035	3.24E−05	3.26E−05	0.994
I-131	2.26E−05	2.09E−05	1.083	2.26E−05	2.31E−05	0.979
Sm-153	2.52E−05	2.28E−05	1.107	2.52E−05	2.59E−05	0.973
Ho-166	6.06E−05	5.63E−05	1.076	6.06E−05	6.17E−05	0.982
Lu-177	1.36E−05	1.22E−05	1.117	1.36E−05	1.40E−05	0.970
Re-186	2.97E−05	2.70E−05	1.097	2.97E−05	3.04E−05	0.975
Re-188	6.78E−05	6.34E−05	1.070	6.78E−05	6.89E−05	0.984
*Auger electron emitters*						
Pd-103	1.54E−06	1.46E−06	1.055	1.54E−06	1.56E−06	0.987
In-111	1.03E−05	9.93E−06	1.035	1.03E−05	1.04E−05	0.993
Sn-117 m	1.74E−05	1.58E−05	1.104	1.74E−05	1.79E−05	0.974
I-123	6.16E−06	5.86E−06	1.050	6.16E−06	6.23E−06	0.989
I-125	4.08E−06	3.86E−06	1.057	4.08E−06	4.13E−06	0.987
Pt-193 m	1.25E−05	1.11E−05	1.129	1.25E−05	1.30E−05	0.967
Pt-195 m	1.82E−05	1.63E−05	1.120	1.82E−05	1.88E−05	0.969

*Radionuclides in parentheses for alpha emitters such as (Po-211) correspond to alpha-emitter decay progeny

**Table 5:** Approximations of $\mathrm{S}\left(\mathrm{LP}\leftarrow \mathrm{LB}\right)$ and $\mathrm{S}\left(\mathrm{LP}\leftarrow \mathrm{LP}\right)$ values for 10 radionuclides with applications to diagnostic imaging using the single-region and dual-region tetrahedral mesh models of the reference adult female liver (AFL)

Radionuclide	*S values (mGy/MBq-s)*					
	Approximations to S(LP ← LB)		Ratio	Approximations to S(LP ← LP)		Ratio
	S(L ← L)	S(LOBV ← LB)		S(L ← L)	S(LOBV ← LOBV)	
*SPECT radionuclides*						
Ga-67	6.03E−06	5.62E−06	1.072	6.03E−06	6.14E−06	0.982
Tc-909m	3.48E−06	3.31E−06	1.050	3.48E−06	3.52E−06	0.989
In-111	1.03E−05	9.93E−06	1.035	1.03E−05	1.04E−05	0.993
I-123	6.16E−06	5.86E−06	1.050	6.16E−06	6.23E−06	0.989
*PET radionuclides*						
C-11	4.85E−05	4.56E−05	1.064	4.85E−05	4.93E−05	0.984
N-13	5.77E−05	5.43E−05	1.062	5.77E−05	5.86E−05	0.984
O-15	7.82E−05	7.39E−05	1.058	7.82E−05	7.93E−05	0.986
F-18	3.57E−05	3.37E−05	1.062	3.57E−05	3.63E−05	0.985
Ga-68	7.73E−05	7.32E−05	1.056	7.73E−05	7.83E−05	0.987
Rb-82	1.34E−04	1.29E−04	1.041	1.34E−04	1.35E−04	0.992

**Table 6:** Approximations of $\mathrm{S}\left(\mathrm{LP}\leftarrow \mathrm{LB}\right)$ and $\mathrm{S}\left(\mathrm{LP}\leftarrow \mathrm{LP}\right)$ values for 22 radionuclides (and 14 additional alpha-emitter decay progeny) with applications to radiopharmaceutical therapy using the single-region and dual-region tetrahedral mesh models of the reference adult male liver (AML)

Radionuclide	*S values (mGy/MBq-s)*					
	Approximations to S(LP ← LB)		Ratio	Approximations to S(LP ← LP)		Ratio
	S(L ← L)	S(LOBV ← LB)		S(L ← L)	S(LOBV ← LOBV)	
*Alpha emitters*						
At-211	1.71E−04	1.54E−04	1.114	1.71E−04	1.77E−04	0.969
(Po-211)	5.16E−04	4.63E−04	1.114	5.16E−04	5.32E−04	0.969
(Bi-207)	2.61E−05	2.57E−05	1.019	2.61E−05	2.62E−05	0.999
Bi-212	1.85E−04	1.68E−04	1.103	1.85E−04	1.91E−04	0.972
(Po-212)	6.09E−04	5.47E−04	1.113	6.09E−04	6.28E−04	0.969
(Tl-208)	7.21E−05	6.96E−05	1.035	7.21E−05	7.26E−05	0.993
Bi-213	3.93E−05	3.65E−05	1.078	3.93E−05	4.01E−05	0.979
(Po-213)	5.80E−04	5.21E−04	1.113	5.80E−04	5.99E−04	0.969
(Tl-209)	6.92E−05	6.64E−05	1.042	6.92E−05	6.97E−05	0.993
(Pb-209)	1.33E−05	1.22E−05	1.095	1.33E−05	1.37E−05	0.974
Ra-223	4.00E−04	3.59E−04	1.114	4.00E−04	4.13E−04	0.969
(Rn-219)	4.69E−04	4.21E−04	1.114	4.69E−04	4.84E−04	0.969
(Po-215)	5.11E−04	4.59E−04	1.114	5.11E−04	5.28E−04	0.969
(Pb-211)	3.11E−05	2.91E−05	1.069	3.11E−05	3.17E−05	0.981
(Bi-211)	4.55E−04	4.08E−04	1.114	4.55E−04	4.69E−04	0.969
(Tl-207)	3.31E−05	3.09E−05	1.068	3.31E−05	3.37E−05	0.981
(Po-211)	5.16E−04	4.63E−04	1.114	5.16E−04	5.32E−04	0.969
Ac-225	4.02E−04	3.61E−04	1.114	4.02E−04	4.15E−04	0.969
(Fr-221)	4.37E−04	3.93E−04	1.114	4.37E−04	4.52E−04	0.969
(At-217)	4.89E−04	4.39E−04	1.114	4.89E−04	5.05E−04	0.969
Th-227	4.14E−04	3.72E−04	1.114	4.14E−04	4.27E−04	0.969
*Beta and positron emitters*						
Sr-89	3.89E−05	3.65E−05	1.064	3.89E−05	3.95E−05	0.983
Y-90	6.13E−05	5.84E−05	1.051	6.13E−05	6.20E−05	0.989
I-124	2.62E−05	2.55E−05	1.029	2.62E−05	2.63E−05	0.996
I-131	1.79E−05	1.67E−05	1.068	1.79E−05	1.82E−05	0.982
Sm-153	1.95E−05	1.80E−05	1.088	1.95E−05	2.00E−05	0.976
Ho-166	4.68E−05	4.41E−05	1.062	4.68E−05	4.76E−05	0.984
Lu-177	1.05E−05	9.59E−06	1.097	1.05E−05	1.08E−05	0.973
Re-186	2.29E−05	2.12E−05	1.080	2.29E−05	2.34E−05	0.978
Re-188	5.25E−05	4.97E−05	1.057	5.25E−05	5.32E−05	0.987
*Auger electron emitters*						
Pd-103	1.21E−06	1.15E−06	1.046	1.21E−06	1.22E−06	0.988
In-111	8.44E−06	8.20E−06	1.030	8.44E−06	8.48E−06	0.995
Sn-117m	1.36E−05	1.25E−05	1.086	1.36E−05	1.40E−05	0.977
I-123	4.98E−06	4.79E−06	1.041	4.98E−06	5.03E−06	0.991
I-125	3.22E−06	3.07E−06	1.048	3.22E−06	3.26E−06	0.988
Pt-193m	9.68E−06	8.74E−06	1.108	9.68E−06	9.97E−06	0.970
Pt-195m	1.42E−05	1.29E−05	1.100	1.42E−05	1.46E−05	0.973

*Radionuclides in parentheses for alpha emitters such as (Po-211) correspond to alpha-emitter decay progeny

**Table 7:** Approximations of $\mathrm{S}\left(\mathrm{LP}\leftarrow \mathrm{LB}\right)$ and $\mathrm{S}\left(\mathrm{LP}\leftarrow \mathrm{LP}\right)$ values for 10 radionuclides with applications to diagnostic imaging using the single-region and dual-region tetrahedral mesh models of the reference adult male liver (AML)

Radionuclide	*S values (mGy/MBq-s)*					
	Approximations to S(LP ← LB)		Ratio	Approximations to S(LP ← LP)		Ratio
	S(L ← L)	S(LOBV ← LB)		S(L ← L)	S(LOBV ← LOBV)	
*SPECT radionuclides*	4.85E−06	4.58E−06	1.059	4.85E−06	4.92E−06	0.985
Ga-67	2.85E−06	2.74E−06	1.040	2.85E−06	2.88E−06	0.991
Tc-99m	8.44E−06	8.20E−06	1.030	8.44E−06	8.48E−06	0.995
In-111	4.98E−06	4.79E−06	1.041	4.98E−06	5.03E−06	0.991
I-123	4.85E−06	4.58E−06	1.059	4.85E−06	4.92E−06	0.985
*PET radionuclides*						
C-11	3.86E−05	3.67E−05	1.052	3.86E−05	3.91E−05	0.987
N-13	4.57E−05	4.35E−05	1.051	4.57E−05	4.63E−05	0.987
O-15	6.15E−05	5.88E−05	1.047	6.15E−05	6.22E−05	0.989
F-18	2.87E−05	2.73E−05	1.051	2.87E−05	2.91E−05	0.987
Ga-68	6.08E−05	5.82E−05	1.045	6.08E−05	6.14E−05	0.990
Rb-82	1.05E−04	1.02E−04	1.033	1.05E−04	1.06E−04	0.994

For each radionuclide, three sets of *S* values were computed: $\mathrm{S}\left(\mathrm{L}\leftarrow \mathrm{L}\right)$ from the single-region liver models, and both $\mathrm{S}\left(\mathrm{LOBV}\leftarrow \mathrm{LB}\right)$ and $\mathrm{S}\left(\mathrm{LOBV}\leftarrow \mathrm{LOBV}\right)$ from the dual-region liver models. As presented previously, values of $\mathrm{S}\left(\mathrm{L}\leftarrow \mathrm{L}\right)$ and $\mathrm{S}\left(\mathrm{LOBV}\leftarrow \mathrm{LB}\right)$ are offered as approximations to the desired value of $\mathrm{S}\left(\mathrm{LP}\leftarrow \mathrm{LB}\right)$, while values of $\mathrm{S}\left(\mathrm{L}\leftarrow \mathrm{L}\right)$ and $\mathrm{S}\left(\mathrm{LOBV}\leftarrow \mathrm{LOBV}\right)$ are offered as approximations to the desired value of $\mathrm{S}\left(\mathrm{LP}\leftarrow \mathrm{LP}\right)$. Again, $\mathrm{S}\left(\mathrm{LP}\leftarrow \mathrm{LB}\right)$ and $\mathrm{S}\left(\mathrm{LP}\leftarrow \mathrm{LP}\right)$ represent, respectively, the absorbed dose to liver parenchyma from radionuclide decays in liver blood (a cross-dose component) and from radionuclide decays in liver parenchyma (a self-dose component).

Ratios of $\mathrm{S}\left(\mathrm{L}\leftarrow \mathrm{L}\right)$ to $\mathrm{S}\left(\mathrm{LOBV}\leftarrow \mathrm{LOBV}\right)$ are shown in the final column on Table 4 for the adult female liver, which quantify the impact one can expect in moving from the single-region to the dual-region liver model in regards to parenchymal self-dose. Average *S* value ratios are shown to be 0.971, 0.980, and 0.981 for the alpha emitters, beta/positron emitters, and Auger electron emitters, respectively, for the therapy-related radionuclides. Thus, one may conclude that the use of the single-region liver model provides an underestimate of liver parenchymal self-dose of only 2–3%. In the fourth column of Table 4, a similar *S* value ratio is given between the single-region and dual-region liver models, but for the cross-dose to liver parenchyma from radionuclide decays within liver blood. Here, the *S* values ratios are on average 1.114, 1.081, and 1.079, respectively, for the same three categories of therapy-related radionuclides. Here, we see that the conventional single-region liver model overestimates the blood cross-dose to liver parenchyma by between ~ 8 and 11%. A similar analysis for the adult male liver model in Table 6 yields average *S* value ratios of 0.975, 0.983, and 0.983 for liver parenchyma self-dose and average *S* value ratios of 1.095, 1.066, and 1.066 for blood cross-dose to liver parenchyma for the same series of therapy radionuclides.

For the imaging-related radionuclides, *S* values ratios for both liver parenchyma self-dose and cross-dose to liver parenchyma from radionuclide decays within the liver blood are displayed in the fourth column and last column of Tables 5 and 7, respectively. *S* values ratios for the cross-dose to liver parenchyma are on average 1.055 and 1.045, ranging from 1.035 to 1.072 and 1.030 and 1.059 for the adult female liver and adult male liver, correspondingly. For the parenchyma liver self-dose, average *S* value ratios of 0.987 and 0.990 were obtained for AFL and AML models, respectively.

### Model limitations

The vascular models created in this study for the AFL/AML models have several limitations. First, pre-capillaries, capillaries, and sinuses are not considered. By excluding these structures, the blood volume of our vascular models accounts for only 15% and 13% of the reference blood volume in the AFL and AML, respectively. Although our models do not account for the total blood volume in the liver, the main arteries and veins of radius ranging from several millimeters to 0.1 mm are explicitly modeled for the arterial, hepatic portal, and hepatic venous blood circulations. Again, our hypothesis has been that it is in these major blood vessels that a majority of blood self-dose, with concomitant reduction in parenchyma absorbed dose, will be realized. Another limitation of our model is that it does not include anastomoses present in real liver vasculature and rather considers that each liver segment has just one entry vessel of each type (HA, HPV, and HV) from which the vascular trees develop.

### Model applications—internal dosimetry

Our research is presently extending this work to other organs of the ICRP mesh-type adult reference phantoms to include the brain, heart, lungs, kidneys, thyroid, and female breasts. Additionally, we are revising the existing mesh-based models of the major arteries and veins already present within the ICRP Publication 145 mesh-type phantoms to include more anatomical realism in their branching, body region location, vessel diameters, and entry to those organs for which new intra-organ vasculature is developed. For internal dosimetry—applied either to diagnostic imaging or to cancer therapy radionuclides—we envision a refinement of *S* values for organ self-dose to now explicitly differentiate decay sites pertinent to radiopharmaceutical tissue localization from those of the radiopharmaceutical while still in general blood circulation. These revisions—by moving from single-region to dual-region organ models—will also refine *S* values for blood as a source region. In fact, values of $\mathrm{S}\left(\mathrm{Target Organ}\leftarrow \mathrm{blood}\right)$ provided in ICRP Publication 133 are generated solely as a blood content weighted average of values of $\mathrm{S}\left(\mathrm{Organ }\leftarrow \mathrm{Organ}\right)$ which themselves are computed using only the single-region organ models within the existing ICRP reference phantoms.

### Model applications—external beam radiotherapy

Another application of these models is in the field of external beam radiotherapy, where there is increasing interest in dose avoidance of circulating lymphocytes during either photon or proton cancer radiotherapy. By declaring circulating blood cells an organ-at-risk (OAR), dosimetric techniques are thus needed to compute dose-volume histograms to circulating blood cells, and to do this, anatomic models as described in this study are needed for Monte Carlo-based simulations of the cancer treatment. In fact, the dual-region model of the adult reference liver presented here has been applied in the study by Xing et al. to explore the dosimetric impact of treatment modality (VMAT and IMRT photon radiotherapy, and both passive scattering and active scanning proton radiotherapy), delivery time, and fractionation schedule on the absorbed dose distributions received by circulating blood cells during individual patient hepatic tumor radiotherapy treatments [40].

## Conclusions

A dual-region liver model that differentiates the organ’s main vasculature and its tissue parenchyma is presented from which SAF values for monoenergetic alpha particles, electrons, positrons, and photons were computed, and then compared to results from the existing single-region liver models of the ICRP mesh-type reference adult phantoms from ICRP Publication 145 [11]. SAF values for the absorbed dose to the liver parenchyma from decay sites in liver blood given by the single-region liver model were shown to exceed those from the dual-region liver model, where blood self-dose is explicitly considered, by 14% for both alpha particles (all energies) and low-energy (< 100 keV) electrons. Similarly, *S* values calculated for therapy radionuclides using the single-region liver model were up to 14%, 12%, and 13% higher than given by the dual-region liver models for alpha emitters, electron/positron emitters, and Auger electron emitters, respectively. In the same way, computed *S* values for SPECT and PET imaging radionuclides using the single-region liver models were up to 7% higher than from the dual-region liver models. The methods presented for intra-organ vascular modeling in the ICRP reference adult male and adult female liver are readily extended to all organs of the reference phantom to permit from an explicit accounting of organ parenchymal dose reduction for short-ranged radiation emissions of the radiopharmaceutical occurring during organ blood transit. The methodology employed in this study thus explicitly allows for the consideration of blood self-dose which is shown to be important for alpha particles (all energies) and for electrons at energies below ~ 100 keV.

## Data Availability

The polygon mesh and tetrahedral mesh models of the reference adult male and reference adult female liver are available upon request to the corresponding author.

## Footnotes

1 See review article by Bolch et al. [5]. Reference phantoms refer to those standardized 50th percentile anatomies defined by the ICRP. Patient-dependent phantoms refer to a library of phantoms that vary by combinations of both total body mass and standing height. Patient-sculpted phantoms refer to the process of matching a specific phantom from a patient-dependent phantom library (closest height/weight) and then deforming (2D or 3D) the outer body contour to match that of the individual patient.

2 http://medapps.emory.edu/surgical-anatomy-of-the-liver/.

3 McNeel, R., & others. (2010). Rhinoceros 3D, Version 6.0. Robert McNeel & Associates, Seattle, WA.

4 We separately computed SAFs for monoenergetic positrons (collisional component only). Even at the lowest energy of 10 keV (where stopping powers maximally differ), percent differences between electron and positron SAFs were less than ~ 0.2%. Electron SAFs were thus employed in the calculation of S values for all positron emitters.
